# Editorial: Evolution, molecular mechanisms and the strategies to combat antimicrobial resistance (AMR): a One Health approach

**DOI:** 10.3389/fcimb.2025.1582613

**Published:** 2025-06-02

**Authors:** Nayeem Ahmad, Ronni Mol Joji, Vineet Kumar, Leonardo Pagani, Mohammad Shahid

**Affiliations:** ^1^ Department of Microbiology, Immunology, and Infectious Diseases, College of Medicine and Medical Sciences, Arabian Gulf University, Manama, Bahrain; ^2^ Department of Microbiology, School of Life Sciences, Central University of Rajasthan, Ajmer, Rajasthan, India; ^3^ Antimicrobial Stewardship Unit, Division of Infectious Diseases, Provincial Hospital of Bolzano, Bolzano, Italy

**Keywords:** antimicrobial resistance, One Health, strategies, antibiotics, molecular mechanisms

## Introduction

**Figure 1:**
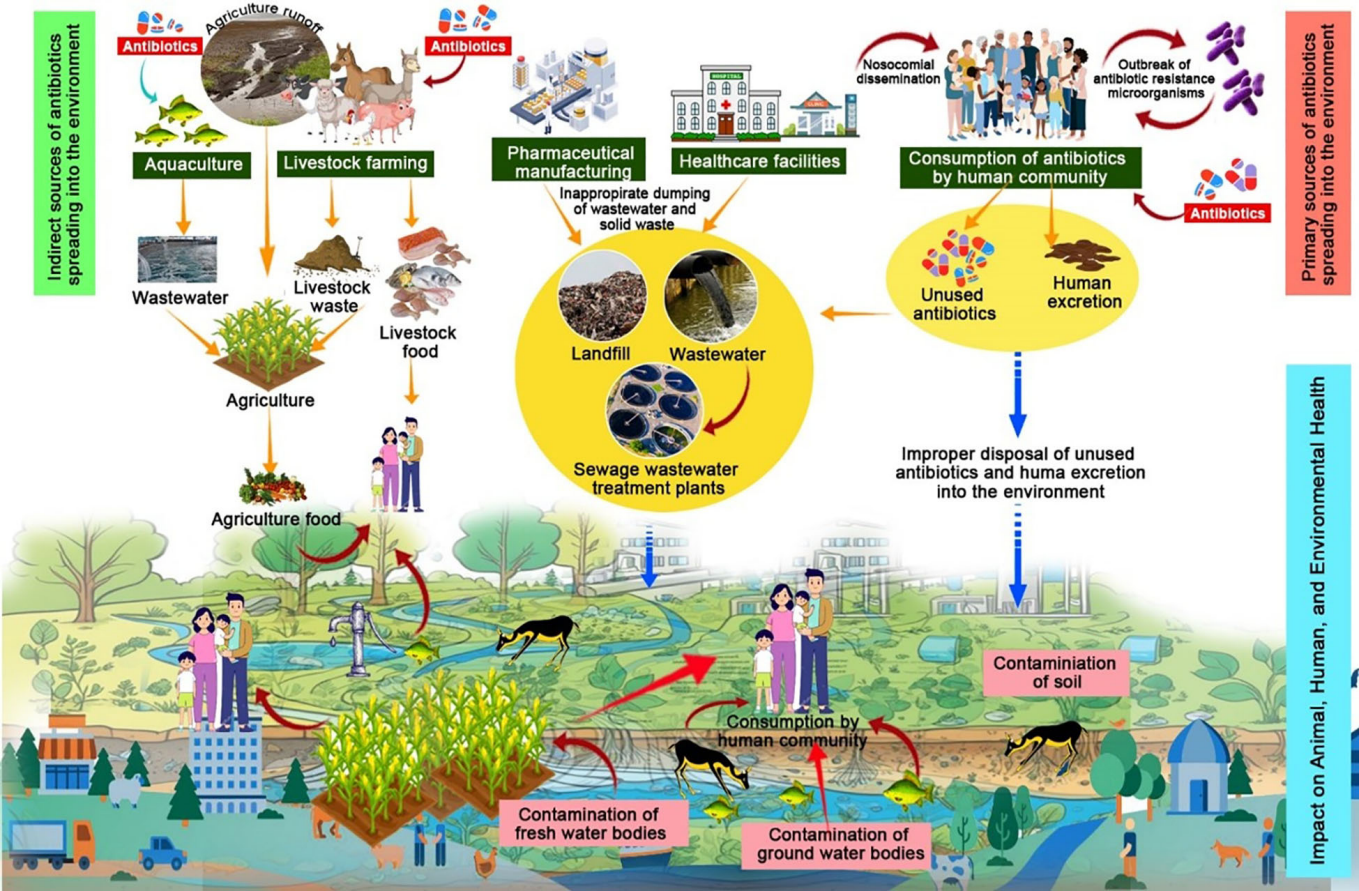
Schematic representation of antimicrobial resistance (AMR) within a One Health approach, emphasizing its interconnected transmission across humans, animals, and the environment.

Antimicrobial resistance (AMR) is a serious global issue that impacts animal, environmental, and human health (Ahmad et al., 2022). AMR occurs when bacteria, viruses, fungi, and parasites resist the effects of antimicrobial agents. This resistance makes the antimicrobial drugs ineffective, leading to infections that are challenging to treat and increases the risk of disease spread, severe illness and death (World Health Organisation, 2023). Overuse and misuse of antibiotics have resulted in drug resistance in various bacteria, making treatments ineffective against certain disease-causing germs (Ahmad et al., 2024). Hence AMR must be addressed from various perspectives to be framed within the One Health paradigm (Collignon and McEwen, 2019). The necessity for a comprehensive strategy to address antimicrobial resistance that considers the health of people, animals, and plants as well as the part the environment plays in mediating the spread of AMR is acknowledged by a One Health approach. This approach advocates the development of collaborative systems for efficiently tracking the establishment and migration of resistant bacteria and resistance genes related to human, animal, and environmental health (World Health Organization, 2014; Franklin et al., 2024). **Figure 1** highlights the role of healthcare, community, agriculture, and environmental factors in the spread of AMR.

To combat AMR effectively, it is essential to understand its evolution, molecular mechanisms, and various available strategies. This includes adopting a one-health approach that addresses AMR across multiple disciplines. In this Research Topic, we aim to present concise and innovative articles describing antibiotic resistance’s evolution and molecular mechanisms, along with potential solutions.

We are passing through a phase that might end up in a sort of pre-antibiotic era due to resistance developed against all classes of antibiotics in bacterial strains, in community and hospital settings (Ahmad et al., 2020). AMR is linked to higher rates of patient death and morbidity, poorer treatment results, and increased expenses for healthcare. According to a recent comprehensive analysis, bacterial AMR was responsible for 4.95 million fatalities in 2019, of which 1.27 million were directly linked to AMR. According to the findings of the Global Burden of Diseases, Injuries, and Risk Factors (GBD) research conducted in 2019, AMR ranked third among all level 3 causes of death in the GBD hierarchy, followed by stroke and ischemic heart disease. AMR was directly responsible for 10.5 fatalities per 100,000 people in East Asia and 27.3 deaths per 100,000 people in sub-Saharan Africa, making the mortality burden in resource-poor environments alarming (Antimicrobial Resistance Collaborators, 2022). The public health threat posed by multidrug-resistant (MDR) bacterial infections has grown globally (Shahid et al., 2023). One of the worrying issues in immunosuppressed patients is the development of antiviral drug resistance, which is a consequence of sustained replication of the virus with prolonged exposure to the drug leading to the selection of resistant strains. The development of rapid clinically relevant diagnoses is focused on the identification of specific viral mutations associated with resistance, which are borne out by targeted phenotypic assays. Key recommendations include balance in drug treatment, improving patient factors, changing therapy based on mechanisms of resistance, and promoting new antiviral drugs (Strasfeld and Chou, 2010).

Another underappreciated aspect of antimicrobial resistance, a global concern, is invasive fungal infections, which represent a serious threat to public health. During a time of significant environmental change on a worldwide scale and growing populations at risk, pathogenic fungi that infect humans are developing resistance to all approved systemic antifungal medications. Strategies for risk reduction are required to minimize the development of resistance in pathogenic fungi (Fisher et al., 2022). Antifungal resistance is growing among fungus, particularly in *Aspergillus* and *Candida* (Etienne et al., 2021). Drug resistance in parasitic infections has a devastating influence on disability-adjusted life year estimates. In fact, it has been reported that drug resistance is on the rise for protozoan parasites (Hanboonkunupakarn and White, 2022) such as *Plasmodium*, *Giardia, Leishmania, Trypanosoma*, or *helminths* (Tinkler, 2020). Drug-resistant *Plasmodium* species are one of the factors impeding the elimination of malaria. *Giardia*, a protozoan parasite that affects both humans and animals worldwide, is linked to rising rates of drug resistance and treatment failures for common medications like metronidazole and albendazole (Picot et al., 2022). The primary causes of the increased burden of AMR are the high prevalence of infectious diseases, insufficient infection prevention and control measures, and the overuse of antimicrobials in the agriculture and human health sectors (Fuller et al., 2022). As a result, the global agenda on antimicrobial resistance has to incorporate the problem of antimicrobial resistance more thoroughly from a One Health perspective (Picot et al., 2022).

## Key approaches and insights from the articles included in this Research Topic

This Research Topic significantly advances our comprehension of the previously mentioned themes outlined above. The 21 important studies make up our Research Topic centred on AMR, its mechanisms, and the potential solutions to combat AMR. In this section focusing on AMR and its various mechanisms, Álvarez-Ainza et al. studied antibiotic resistance in ESKAPE microorganisms, and they noted high resistance to cephalosporins and ciprofloxacin among *Klebsiella pneumoniae* and *Acinetobacter baumannii.* Additionally, Uruen et al. also studied AMR rates and the genetic origin in invasive *Streptococcus suis* isolates recovered in Spain from 2016 to 2021. They found high resistance rates (>80%) for tetracyclines, marbofloxacin, lincosamides, and spectinomycin. PCR screening and whole genome sequencing revealed 23 AMR genes, including four novel ones (*aph(*2’’)-IIIa, *erm*(47), *tet*(T) and *apm*A. Several genes were found on mobile genetic elements. Furthermore, mutations in genes encoding target enzymes (*pbp1a, pbp2b, pbp2x, mraY, gyrA, parC, and dhfr*) were found. The study findings showed that *S. suis* is a significant contributor to AMR spread in both veterinary and human infections. Hence, they stated that AMR control in *S. suis* should be considered as part of a One Health approach in places with substantial pig production.

Huang et al. reported the presence of MRSA strain SA2107 which belongs to the global ST45 lineage, from a 5-year-old child in China. Its genome, approximately 2.9 Mb, included two plasmids, prophages, integrative elements, and insertion sequences and it carried the SCCmec IVa (2B)-t116 type. It also carried beta-lactam resistance genes and virulence factors, with prophages harboring additional virulence genes. Additionally, Patil et al. study also analyzed 29 ESBL-producing *E. coli* ST410 isolates from pediatric patients. The most prevalent ESBL gene was *bla*_CTX-M_. *FimH, papC*, and *hlyA* were the common virulence genes present in these isolates. The isolates also harbored diverse plasmids and serotype analysis identified several types, including O1:H7 and O2:H1. Interestingly Petakh and Kamyshnyi conducted a study on AMR mechanisms in *L. interrogans* serovars and they found 32 genes linked to AMR throughout the analysis, 20 of which were important genes that were consistently present in most strains. Remarkably, they also discovered diverse resistance pathways in serovar Pomona by identifying discrete efflux pump systems. This study has expanded the knowledge of resistance mechanisms in serovar Pomona by identifying the presence of distinct efflux pump systems. The study concluded that it is crucial to implement specialized intervention plans and work together with environmental scientists, veterinary specialists, and human healthcare providers to address the intricate dynamics of leptospirosis and its consequences for antibiotic resistance.

Given the scarcity of available treatments, the development of ceftazidime-avibactam (CZA) resistance in carbapenem-resistant *Klebsiella pneumoniae* (CRKP) is a serious issue. Chen et al. assessed carbapenem resistance gene transferability using conjugation procedures and whole-genome sequencing (WGS) on 10 CRKP isolates. They discovered that the blaKPC-145 gene was found on an untransformable IncFII-type plasmid that was 148,185 bp long. When carbapenem was used to treat a *K. pneumoniae* infection that produced KPC-145, the production of KPC-2 was reversed. According to WGS analysis, there were 14 SNPs, and all isolates belonged to the ST11-KL47 group. Their results showed that during CZA treatment, frequent testing for carbapenemase genotype and antibiotic susceptibility is crucial. In another work by Lin et al. a novel chromosomal aminoglycoside resistance gene, designated *aph*(3’)-Ie, was identified in a rabbit isolate *C. gillenii* DW61.

In addition to bacterial resistance, fungal resistance such as azole-resistant *Aspergillus* or echinocandin-resistant Candida is a growing concern, particularly in immunocompromised patients. Studies on Nakaseomyces glabratus (Candida glabrata) underline the persistence of antifungal resistance and its clinical challenges. Ksiezopolska et al. investigated the stability of drug resistance in *Nakaseomyces glabratus* under non-selective conditions. They observed that anidulafungin resistance had higher stability as compared to fluconazole resistance. Also, in non-selective conditions, they found a build-up of novel mutations in resistance-associated genes that had previously undergone alteration, which might indicate a compensatory role. They concluded that the persistence and spread of drug-resistant clinical epidemics are significantly impacted by the long-lasting nature of acquired resistance, especially to anidulafungin. The persistence and spread of antifungal resistance in *Nakaseomyces glabratus* highlight the urgent need for surveillance and innovative treatments, reinforcing the importance of an integrated One Health approach to address AMR across all microbial domains.

Individuals with HIV/AIDS can be affected by antimicrobial resistance (AMR) in two key ways. First, HIV patients may develop resistance to antiretroviral drugs, especially when medication adherence is inconsistent. This form of resistance, known as HIV drug resistance, can arise within the patient and may also be transmitted from mother to child during pregnancy or between sexually active individuals who are infected (Global Antibiotic Research and Development Partnership). Drug resistance in HIV-infected people needs to be tracked to detect the drug resistance pattern, revise treatment plans, and establish a scientific basis for AIDS prevention and control. A study of HIV-positive patients in Meizhou was carried out by Liu et al. They found that reactivity with seven bands (p24, p31, gp41, p51, p66, gp120, and gp160) was the most prevalent pattern instead of the full-band pattern seen elsewhere. The most commonly absent bands included p55, p39, and p17. Additionally, they also noted that patients in the middle to advanced stages of the infection had lower levels of the p17 antibody. They also observed that most resistant mutations were linked to nucleoside reverse transcriptase inhibitors and non-nucleoside reverse transcriptase inhibitors. Just one case had a mutation resistant to protease inhibitors, indicating that the inclusion of protease inhibitors in the treatment plan would benefit the patients in Meizhou.

Antimicrobial-resistant bacterial prevalence has reached an unprecedented level globally, posing a silent pandemic threat and thus demands immediate action. Antimicrobial-resistant infections have very few therapeutic options, thus leading to high morbidity and mortality and substantial financial costs. Monitoring and surveillance, reducing the use of antibiotics in food animals and over-the-counter medications, providing access to high-quality, reasonably priced medications, vaccinations, and diagnostics, and enforcing the rules and regulations are all immediate measures to prevent AMR. An immediate cooperative action must be taken immediately by the national and international organizations so that we don’t step into the post-antibiotic era (Salam et al., 2023).

Zuberi et al. in their review article, addresses biofilm and its role in antibiotic resistance, and the challenges faced in treating biofilm-related infections. This study showcases cutting-edge therapeutic strategies to fight drug-resistant biofilm infections, such as CRISPR/Cas9 gene editing, transcriptomics, metabolomics, and bioinformatics. The review explains future hopes for better therapies while highlighting current research in innovative anti-biofilm agents and biofilm prevention techniques. Another article by Al-Fadhli and Jamal attempted to describe the most recent advancements in gene editing technology, with a focus on CRISPR/Cas9. The study described the potential of the innovative strategies, and their advantages and disadvantages when compared to more conventional methods. Overall, the review aimed to guide readers on the efforts to manage and prevent the emergence of AMR with an appraisal of their challenges. According to Liu et al., 1H-Pyrrole-2,5-dicarboxylic acid, which was initially isolated from P. tephropora FF2, an endophytic fungus of A. catechu L., may prevent *P. aeruginosa* PAO1 from forming biofilms and virulence factors. They observed that this acid works as an antibiotic accelerant against *P. aeruginosa* PAO1 infection and demonstrates strong quorum-sensing inhibitor action. Additionally, a study by Oves et al. focused on pyocyanin synthesis by *P. aeruginosa*, which was found to be associated with anticandidal activity. The minimum inhibitory concentration (MIC) of the bacterial extract against *Candida albicans* was 50 μg/ml, whereas the pyocyanin-based MIC was marginally lower at 38.5 μg/ml. They also noted that interactions between *P. aeruginosa* and *Candida albicans* resulted in candida destruction. They observed that this acid works as an antibiotic accelerant against *P. aeruginosa* PAO1 infection and demonstrates strong quorum sensing inhibitor action.

Magnolol is a traditional Chinese medicine that is shown to exhibit antibacterial properties. Qiao et al. investigated the mechanism of action of magnolol against *Mycoplasma synoviae* using metabolomics research. According to this investigation, magnolol exhibited an outstanding inhibitory effect against a range of mycoplasmas. *In vitro*, magnolol demonstrated dose-dependent suppression of *Mycoplasma synoviae* growth and biofilm formation. This explains the possibility of the use of magnolol compound to treat mycoplasma infections. Almuhanna studied the fragrant desert shrub Ducrosia anethifolia, which is used in traditional Saudi medicine. In both *in vitro* and *in vivo* tests, a methanolic extract of this plant showed more antibacterial action against methicillin-resistant *Staphylococcus aureus* (MRSA) than MDR-*P.aeruginosa*. Additionally, the extract-derived ointment formulation demonstrated improved diabetic wound healing. It also reduced the microbial load of both pathogens in the wounded tissue.

Metallic nanoparticles have recently come to light as viable options for addressing the risk of antibiotic-resistant infections. Zubair et al. Investigated the effectiveness of Artemisia vulgaris-biosynthesized tin oxide nanoparticles (AvTO-NPs) against the virulence factors and biofilms of drug-resistant *Candida albicans* strains. The researchers observed that AvTO-NPs showed MICs ranging from 1 mg/mL to 2 mg/mL. In the test strains at the corresponding 1/2xMIC, AvTO-NPs markedly reduced the synthesis of exopolysaccharide by 69%–86.3%, germ tube formation by 72%–90%, cell surface hydrophobicity by 68.2%–82.8%, and biofilm formation by 54.8%–87%. This study also noted that mature biofilms were considerably disrupted by these NPs. Additionally, a study by Abdikakharovich et al. demonstrated that zinc oxide nanoparticles showed a significant ability to lessen the dermatitis-causing effects caused by *Staphylococcus aureus* following a one-week therapeutic intervention in murine models.

A significant concern stemming from the SARS-CoV-2 infection is the potential long-term spread of AMR in acute care settings. This risk arises from increased patient exposure to antimicrobials, which are often used suboptimally or inappropriately. Evaluating both the immediate and long-term impacts of the SARS-CoV-2 infection on antimicrobial usage and the emergence of drug-resistant infections is very crucial (Rawson et al., 2020). SARS-CoV-2 infection in humans is due to the presence of its spike protein which plays a critical role. Human proteases, such as furin protein, break the S subunit spike protein, which is a crucial first step in the internalisation of the virus into a human host. Tiwari et al. tried to inhibit furin by utilising phytochemicals. They screened 408 naturally occurring phytochemicals with antiviral qualities from different plants against the furin protein and also ran molecular docking and dynamics simulations. They observed that by targeting furin, the natural chemicals robustaflavone, withanolide, and amentoflavone may have therapeutic potential for the management of SARS-CoV-2.

A review paper by Kumar et al. concentrated on how antibiotic adjuvants work in concert to combat multidrug-resistant bacteria. First, they can make current antibiotics more effective against resistant strains. Second, adjuvants can improve the antibiotic penetration capacity into bacterial cells, increase their stability, or block the efflux pumps. Efflux pump inhibitors, resistance-modifying agents, and compounds that disrupt bacterial biofilms are among the many kinds of antibiotic adjuvants that have been studied.

The nations can characterize the geographic and temporal patterns and scale of AMR by conducting regular national AMR surveillance. This data is crucial for directing local empirical antibiotic treatments and for developing and improving public health policies and initiatives targeted at lowering AMR (van der Kuil et al., 2017). The purpose of a study by Mao et al. was to determine the main variables affecting Cambodia’s adoption of a laboratory-based AMR surveillance system. The main obstacles to putting in place an efficient surveillance system, according to key informants, were lack of manpower, limited capacity to gather representative data, an unsustainable finance system, and insufficient access to laboratory supplies. Issues in data analysis and quality assurance were found in the laboratory assessments, which could limit the facility-level use of aggregated data and lower the quality of data transmitted to the surveillance system.

According to Kim et al. Kor-GLASS, established in South Korea, keeps an eye on AMR using a quality control centre that guarantees accurate data. Despite a decrease in the usage of human antibiotics, resistance rates have varied since 2020, underscoring the necessity of improved non-human drug sales monitoring. They reported that to address AMR, the project now takes a “One Health” stance, focusing on integrated management across humans, animals, and the environment to address AMR, the project now takes a “One Health” stance, focusing on integrated management across humans, animals, and the environment.

## Conclusion

To conclude, the studies in this Research Topic highlight the complexity of AMR. Highlights of the Research Topic include various AMR mechanisms, potential AMR solutions including antibiotic adjuvants, the use of nanoparticles, biofilm-targeted therapeutic ways, and natural antimicrobials. To properly monitor and control AMR, surveillance systems such as Kor-GLASS highlight the significance of integrated “One Health” approaches. Immediate action is essential to combat AMR including lowering antibiotic abuse, improving infection control, and encouraging interdisciplinary teamwork. In the battle against AMR, drug resistance to fungi, viruses, and parasites must be addressed since these microbial groups play a major role in drug-resistant illnesses that pose a threat to global health. To safeguard the health of human, animals, and the ecosystem, a comprehensive and cohesive One Health approach must be followed to combat such forms of resistance.
